# Advancing Human-Centered AI in Clinical Decision Support: Sociocognitive Human-in-the-Loop Study in HIV Care

**DOI:** 10.2196/91620

**Published:** 2026-07-31

**Authors:** Dezhi Wu, Valerie Vera, Sai Krishna Revanth Vuruma, Lucas Aust, Bharat Sowrya Yaddanapalli, Jiaxuan Zhang, Rithika Markanti, Jiajia Zhang, Xiaoming Li, Sharon Weissman, Bankole Olatosi

**Affiliations:** 1 Department of Integrated Information Technology, University of South Carolina, 550 Assembly Street, Columbia, SC, 29208, United States, 1 803 777 4691; 2 Department of Epidemiology and Biostatistics, Arnold School of Public Health, University of South Carolina, Columbia, SC, United States; 3 Department of Health Promotion, Education, and Behavior, Arnold School of Public Health, University of South Carolina, Columbia, SC, United States; 4 Floyd School of Medicine, University of South Carolina, Columbia, SC, United States; 5 Prisma Health, Columbia, SC, United States; 6 Department of Health Services, Policy, and Management, Arnold School of Public Health, University of South Carolina, Columbia, SC, United States

**Keywords:** clinical decision support systems, AI, CDSS design, HIV, HIV care, human-in-the-loop, human-computer interaction, human-centered AI

## Abstract

**Background:**

AI-powered clinical decision support systems (CDSS) have shown promise in improving prediction, monitoring, and treatment optimization across clinical domains, including HIV care. However, translating AI outputs derived from electronic health records into clinically meaningful, trustworthy, and actionable decision support remains challenging, underscoring the need for more human-centered and socioecologically grounded CDSS design.

**Objective:**

This study aimed to explore how we can effectively translate the outputs of machine learning models based on HIV electronic health records into a real AI-powered CDSS for HIV care. Using the human-in-the-loop method, we engaged a set of stakeholders, including HIV physicians, nurse practitioners, infectious disease pharmacists, social workers, and case managers. Stakeholders interacted with an AI-powered CDSS prototype to identify barriers and challenges to adoption, as well as to inform a more holistic and context-aware AI-powered CDSS design.

**Methods:**

We conducted a field study at Prisma Health in South Carolina that included pre- and postsurveys, interactive usability testing sessions, think-alouds, and in-depth interviews with 16 clinicians providing HIV care between March and September 2025. We analyzed survey responses using descriptive statistics, and then transcribed and analyzed think-aloud and interview data using an etic and emic approach.

**Results:**

Clinicians identified multiple challenges and design considerations for AI-powered HIV CDSS, demonstrating that clinician-AI interaction is inherently sociotechnical and embedded across multiple socioecological levels. While clinicians relied on familiar clinical indicators as cognitive anchors for interpreting AI predictions, they emphasized that social determinants of health were central to their own risk assessment and clinical decision-making. Additionally, clinicians' trust in AI is conditional and develops over time, with explainability and actionability emerging as critical factors for translating predictions into meaningful clinical interventions.

**Conclusions:**

Findings highlight the need to move beyond technically accurate predictions toward AI-powered CDSS designs that align with clinicians’ cognitive practices and socioecological realities of HIV care. By extending a sociocognitive framework through empirical grounding in HIV clinical practice, this study offers design insights for developing AI-powered CDSS that are trustworthy, context-aware, and capable of supporting actionable decision-making in HIV care settings and beyond.

## Introduction

### Background

Today’s AI and machine learning (ML) technologies are redefining clinical decision support systems (CDSS), offering new opportunities to enhance diagnostic accuracy, risk stratification, treatment optimization, and population health management. Prior and ongoing research studies on AI-powered CDSS have demonstrated improvements in workflow efficiency, predictive performance, and personalized care in multiple health care domains [1-4]. However, the integration of AI into clinical practice remains contingent on factors such as explainability, clinical relevance, usability, trust, and alignment with real-world workflows [5-7].

HIV care presents a particularly compelling case for AI-powered decision support. The HIV care continuum, spanning testing, diagnosis, medication adherence, retention, treatment management, and long-term comorbidity monitoring, generates rich longitudinal clinical and laboratory data. However, clinicians face complex decision-making demands involving adherence support, regimen optimization, comorbidity management, and interpretation of rapidly evolving viral resistance profiles. Traditional rule-based CDSS integrated into electronic medical records have already demonstrated measurable benefits for HIV screening, test ordering, follow-up, and retention, especially in resource-limited settings [8-12].

AI and ML approaches extend these capabilities by enabling the prediction of HIV drug resistance, treatment interruption, virologic failure, mortality, and comorbidities using genomic, laboratory, and electronic health record (EHR) data [13-17]. AI also enhances HIV testing and prevention through risk stratification, rapid test interpretation, automated self-testing support, and triaging for pre-exposure prophylaxis [18,19].

AI-powered CDSS have expanded rapidly across clinical domains, improving diagnosis, prognosis, and treatment decisions using structured and unstructured clinical data [2,20-22]. ML models, such as neural networks, random forests, gradient boosting, and natural language processing systems, have shown strong performance for laboratory interpretation, infection management, imaging analysis, and complex multimodal prediction tasks [23,24].

Yet, significant challenges remain despite strong technical performance [5-7,25]. Clinicians frequently report concerns about algorithmic transparency, alignment with clinical judgment, autonomy, data quality, and contextual fit issues that are amplified in HIV care due to stigma, resource constraints, and fragmented care systems [26-28]. Privacy-preserving analytic frameworks, such as secure multiparty computation, illustrate the need for ethical, secure data sharing in HIV contexts [29]. Implementation studies emphasize the importance of leadership support, EHR integration, clinician involvement in design, and alignment with existing organizational processes [30-32]. Evaluation frameworks, such as DECIDE-AI [33], call for prospective, iterative assessment of usability, safety, and workflow impact [34].

Choudhury’s [35] sociocognitive conceptual framework offers a more holistic lens for understanding clinician-AI interactions, integrating systems thinking, the Technology Acceptance Model [36], expectancy theory [37], and distributed cognition. This perspective highlights that AI adoption depends not only on model performance but also on clinicians’ expectations, cognitive load, trust, shared mental models, and ecological validity within routine practice. While existing sociocognitive frameworks have contributed to our understanding of clinician-AI interaction by foregrounding factors such as trust, cognitive workload, and situation awareness, they have largely conceptualized these interactions at the level of individual cognition without evidence-based research. As a result, the current sociocognitive frameworks may limit our view on how broader socioecological and systems-level dynamics shape clinicians’ interpretation and use of AI, particularly in stigmatized care contexts such as HIV, and its feasibility in HIV care in practice.

Although evidence for AI applications in HIV care and for AI-powered CDSS effectiveness is rapidly expanding, there remains a significant need for integrative work that links HIV-specific AI capabilities with CDSS design and implementation evidence, as well as sociotechnical and human factors frameworks. Such synthesis is essential for informing the design of AI-powered HIV CDSS that is accurate, trustworthy, ethically grounded, and feasible across diverse clinical environments. In response, this work significantly extends Choudhury’s [35] sociocognitive framework by offering an empirically grounded, prescriptive framework that illustrates how clinician-AI interactions in HIV care are embedded within broader socioecological systems that actively shape how clinicians interpret, trust, and translate AI-generated outputs into clinical actions. This work is primarily guided by the following overarching research questions (RQs):

- RQ1: What key variables are needed to design a useful AI-powered HIV CDSS?
- RQ2: What are the barriers to, challenges of, and recommendations for creating a holistic, trusted, and engaging AI-powered HIV CDSS system?
- RQ3: What AI design features are needed to provide valuable input into HIV care clinicians’ decision-making processes? Why?

### AI Across the HIV Care Continuum

AI and ML have been increasingly applied across HIV prevention, diagnosis, and treatment. AI-based tools for HIV testing improve the sensitivity and specificity of rapid test interpretation, support mobile self-testing, and reduce false-positive and false-negative errors [19]. Risk prediction models identify candidates for HIV testing, linkage, and pre-exposure prophylaxis [17,18].

In treatment, AI platforms such as DeepHIV and geno2pheno leverage genomic datasets to predict drug resistance and support regimen selection [13]. ML models predict treatment interruption, adherence risk, virologic failure, CD4 lymphocyte trajectory, mortality, and comorbidity onset [14-16]. Systematic reviews highlight strong accuracy but identify gaps in external validation, interpretability, calibration, and fairness [14,15].

### HIV-Specific CDSS

Traditional rule-based HIV CDSS embedded in electronic medical records have shown benefits in increasing HIV screening rates, supporting test ordering, detecting missed appointments, improving follow-up, and enhancing CD4 lymphocyte monitoring [8-12]. HIV-ASSIST demonstrated high concordance with expert prescribing, underscoring its potential as a supportive and educational tool [38].

Provider perceptions highlight enthusiasm for CDSS but emphasize the need for usable interfaces, reliable data, contextual sensitivity, and preservation of clinical autonomy [26,27]. These findings align with broader AI-powered CDSS research demonstrating the centrality of trust, explainability, and clinician-AI collaboration [5,25,39].

### Sociotechnical, Ethical, and Human Factors Considerations

Ethical and legal concerns, such as privacy, bias, accountability, and transparency, are pronounced in HIV contexts [19,28]. Privacy-preserving computation frameworks support safe model development but require further work to ensure scalability and usability [29]. This study is primarily informed by Choudhury’s [35] descriptive framework aimed at understanding the dynamic and complex interactions between clinicians and AI systems and providing evidence-based research in clinical settings to ensure the ecological validity of AI integration. The integrative framework uses a systems thinking approach, combining multiple descriptive human factors models to understand clinician-AI interactions in decision-making. As such, the framework constitutes a sociocognitive approach that extends theories of distributed cognition.

Choudhury’s [35] conceptual framework focuses on the interaction between AI and clinicians by emphasizing clinicians’ cognitive functions and perceptions regarding AI by highlighting several crucial factors that influence their intention and willingness to use AI. These factors include trust in AI, patient safety, expectancy, cognitive workload, situation awareness, and perceptions of AI. *First,* the framework acknowledges that clinicians’ trust in technology, which is generally associated with reliability and performance, is essential for AI acceptance. For AI systems specifically, trust stems from clinicians’ confidence that the system will rarely make errors, as achieved through system training, exhaustive testing, safety measures, and standards. *Second,* patient safety emphasizes how clinicians may be misled into making wrong decisions if they misinterpret AI outputs, putting patient safety at risk. Therefore, safety involves minimizing both risk and epistemic uncertainty. Expectancy, then, includes both performance and effort expectancy, suggesting that a clinician’s decision to follow an AI-derived recommendation is dependent on their belief that the decision will lead to an intended outcome (eg, patient safety). Cognitive workload emphasizes the mental burden or strain imposed on clinicians by integrating the AI system into their work environment. Situation awareness, in contrast, captures clinicians’ fundamental understanding of the current clinical situation, which is affected by how the system presents relevant information. *Finally*, perceptions of AI include clinicians’ perceptions regarding technology itself and their concerns about who is responsible for faulty AI outcomes, with a lack of accountability a significant hindrance to AI adoption.

As Choudhury’s [35] proposed framework is descriptive, we leverage it to inform the development of a prescriptive framework for achieving optimal AI-clinician interactions. Building on this foundation, we extend Choudhury’s [35] framework by situating clinician-AI interaction within a socioecological and systems thinking perspective. Through empirical grounding, we aim to determine why clinicians use or reject AI in clinical settings, focusing specifically on HIV care settings. By understanding the dynamic influence of factors such as trust, cognitive workload, and situation awareness, we aim to move from a descriptive account of clinician-AI interaction toward a prescriptive, iterative human-in-the-loop AI design-oriented framework that reflects the lived realities of HIV clinical practice.

In the sections that follow, we reported a field study that we conducted at a Prisma Health HIV clinic in South Carolina from March to September 2025 and detailed the process of using a human-in-the-loop approach to engage HIV care clinicians in iterative AI-powered HIV CDSS design and usability sessions and system implementation. We then reported our survey, think-alouds, and interview data analysis results and recommend intelligent user interface (UI) features.

## Methods

### System Architecture

We used a binary Random Forest (RF) model [40] in combination with Local Interpretable Model-Agnostic Explanations (LIME) to generate the Explanation view for patients. We used inner cross-validation and grid search to identify the best parameters for the RF model from the number of trees set to grow from 500 to 1000, the number of variables randomly sampled as candidates at each split from 5 to 15, and the maximum number of terminal nodes from 5 to 30. For each iteration, the binary RF was tuned using the same training set. The best parameter combination was used to fit the RF model.

The RF model was trained in a deidentified cohort of longitudinal EHR clinical data of all people living with HIV in South Carolina collected from multiple health systems [41]. The sample dataset of patients with HIV used to generate prototype visualizations was extracted from this larger deidentified dataset. The dataset contained features ranging from sociodemographic information (eg, age, gender, race, HIV risk exposure level, and residential type) and baseline HIV markers (eg, initial CD4 lymphocyte count, initial viral load, and time to initial viral suppression) to viral load indicators (eg, viral load, viral suppression, viral rebound, viral blips, number of rebounds after initial suppression, and proportion of time spent with viral load ≤500 copies/mL) and HIV care cascade factors (eg, linkage to care, retention in care, number of visits, comorbidity history, mental health history, and substance use history).

The RF model was evaluated using classification metrics such as *F*_1_-score, area under the curve, sensitivity, and specificity in different lag time windows spanning 4 months each. The Lag 3 RF model, using 12 months of historical information to predict the subsequent 4-month window, achieved an area under the curve of 0.851, a sensitivity (recall) of 0.897, a specificity of 0.730, and an *F*_1_-score of 0.870 [40]. This model was used along with LIME to generate a static Explanation view for the 3 selected patients. The RF model was not actively running during the evaluation sessions.

### Initial AI-Powered HIV CDSS Prototype Design

We designed the initial AI-powered HIV CDSS prototype to better represent patterns and trends in patient data for clinicians. The tool is meant to be an auxiliary that the clinicians could refer to while diagnosing a patient or examining a patient’s progress with respect to their treatment plan. Clinicians currently use Epic, a leading large-scale EHR system used by hospitals and health care providers, which contains all patient information. However, the Epic module used by Prisma Health has a UI that presents an extensive amount of patient data in long table visual aids. Hence, our motivation was to design the prototype with easily understandable visual representations of data, such as graphs and explainability, to be augmented to the existing Epic workflows [42].

**Figure 1.**
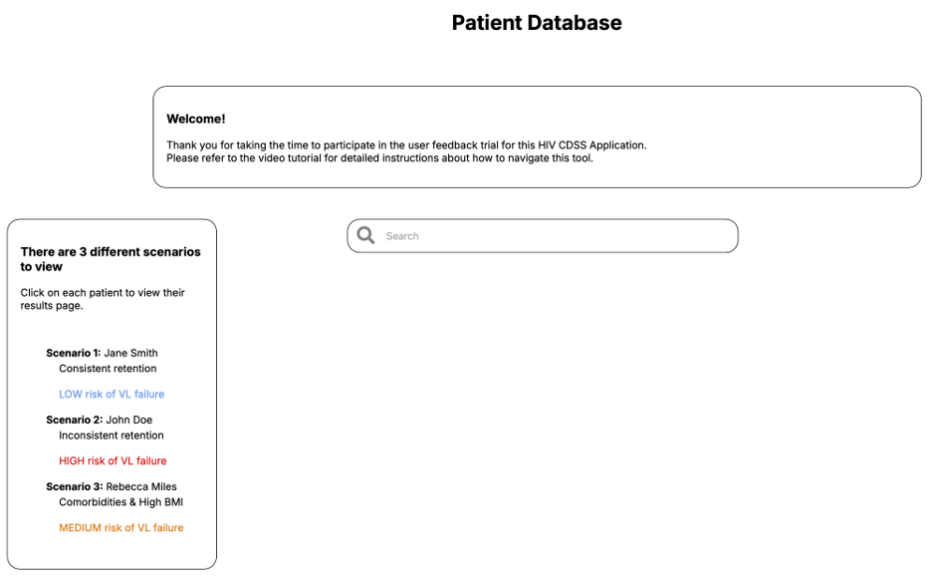
Snapshot of the AI-powered HIV clinical decision support systems prototype showing the patient database interface, including a searchable patient list with 3 clinical scenarios.

**Figure 2.**
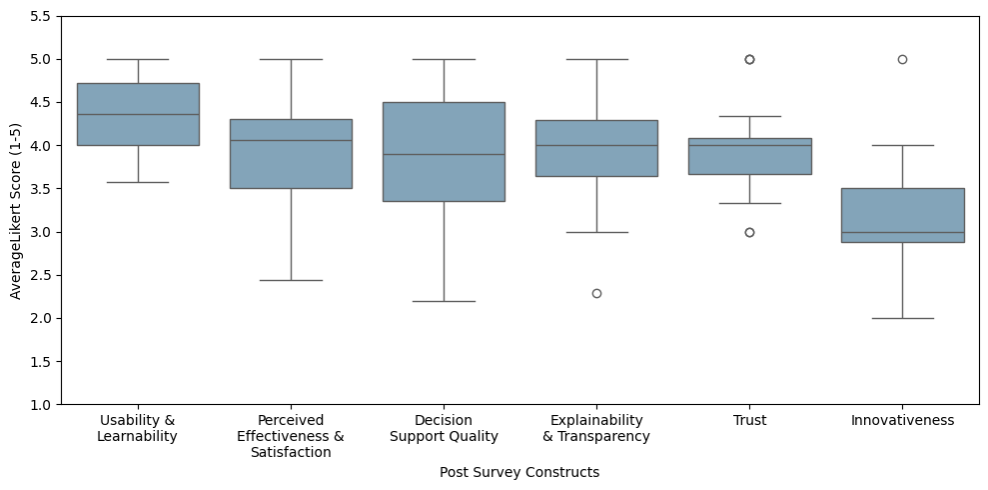
Overall theme-level sentiment toward AI-powered HIV clinical decision support systems prototype across survey constructs.

We used a sample dataset of patients with HIV, which contained patient demographics, viral indicators, such as viral load, viral blip, viral suppression, and viral rebound, and laboratory results, such as CD4 lymphocyte count. After initially interacting with our HIV care clinicians, the viral indicators were identified as the essential data points needed when assessing the condition of a patient because these viral indicators are tested regularly when patients with HIV visit the clinic. As such, in our AI-powered HIV CDSS prototype, these key patient data were represented in a stacked line graph, plotted against patient visit timelines on the x-axis (see snapshots in Figure 1 below and in Figures S1-S3 in Multimedia Appendix 1). The visit timelines shown on the x-axis used numbered indices instead of more familiar timestamps. This lack of legibility may have resulted in lower average scores for the explainability and transparency theme shown below in Figure 2. Supporting demographic information, substance use, and mental health history were highlighted to the left of the graphs, and supporting health indicators, such as BMI, were displayed in a smaller table to their right [42].

**Figure 3.**
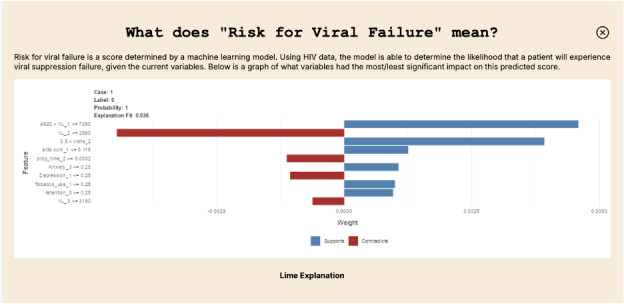
Local Interpretable Model-Agnostic Explanations model for AI explainability of a patient’s predicted risk of viral failure.

The prototype presented to study participants was a static demonstration mockup in which 3 illustrative patient cases were prepopulated with representative risk levels (low, medium, and high) to support clinician feedback. The progress bar displaying each patient’s risk level was designed to reflect the output that would be generated by the RF model in a live deployment; however, the RF model was not actively running during the evaluation sessions. As such, the patient’s risk level in the prototype is a static representation and should not be inferred as the predicted risk level for the respective patient information, which we communicated to participants when they interacted with the prototype. The progress bar is filled with a red color when the risk level is above 67% to convey that this is a high-risk patient for viral failure. Similarly, orange represents a medium risk above 33%, and blue represents a low risk below 33%. To improve interpretability for clinicians, we used the LIME model [43] to highlight the features that influenced the model’s decision when predicting a risk level [44] (Figure 3). These explanations were generated using an RF model adapted from previous research [40,41].

The prototype was implemented primarily using D3.js, enabling interactive and dynamic visualizations within a web-based interface. We also conducted early testing sessions with our HIV care clinicians to gather qualitative feedback on usability and data visualization clarity, which informed plans for subsequent on-site interviews and iterative design sessions to further improve our HIV CDSS design and implementation. Overall, the initial prototype established a foundation for a more clinician-centered CDSS by merging predictive modeling outputs with transparent visualization and contextual explanations.

### Ethical Considerations

This study was reviewed and approved by both the University of South Carolina (USC) and Prisma Health’s Institutional Review Boards under expedited review procedures for research involving human subjects (approval Pro00135845 at USC and approval 2238394‐1 at Prisma Health). We informed participants of the study’s purpose, procedures, the voluntary nature of participation, and their right to withdraw at any time without penalty. Participation was fully voluntary, and participants did not receive any compensation. All participants provided informed consent prior to participation in the study. To protect participant privacy, we deidentified all data prior to analysis and assigned participants unique study identifiers, which we used to refer to participants in the subsequent sections.

### Data Collection and Sample

We recruited 16 HIV care clinicians at Prisma Health to participate in the study between March and September 2025. Participants included physicians (n=4, 25%), nurse practitioners (n=2, 12.5%), infectious disease pharmacists (n=3, 18.75%), social workers (n=2, 12.5%), registered nurses (n=1, 6.25%), medical assistants (n=1, 6.25%), clinical counselors (n=1, 6.25%), outreach supervisors (n=1, 6.25%), and case managers (n=1, 6.25%). Table S1 in Multimedia Appendix 2 indicates their demographic information, job roles, trust in AI, and suggested AI features for the HIV CDSS.

Participants first completed a presurvey that captured self-reported demographic information and familiarity with Epic software. After completing the presurvey, participants reviewed a short tutorial video that demonstrated the initial HIV CDSS prototype design. Participants then interacted with each of the 3 use cases, including low-, medium-, and high-risk patients with HIV in the initial prototype while engaging in a think-aloud protocol, designed for participants to verbalize their thought processes. Following the testing, participants completed a postsurvey that focused on usability and learnability, perceived effectiveness and satisfaction, decision support quality, explainability and transparency, and trust in the prototype, as well as participants’ innovativeness (ie, adoption intention of emerging technologies; Table S2 in Multimedia Appendix 2 details postsurvey constructs and questions).

All participants then engaged in semistructured interviews and interactive usability testing sessions. The usability sessions used simulated patient cases representing varying levels of predicted risk. At the beginning of each session, we informed participants that the cases reflected hypothetical patient information, and we reiterated this framing as needed to maintain clarity regarding the nature of the scenarios. The subsequent interview protocol expanded on the prototype’s usefulness, ease of use, and explainability, as well as participants’ trust in the prototype’s outputs and participants’ perceptions of AI (Multimedia Appendix 3). Interviews averaged between 50 and 75 minutes and were audio recorded with participants’ consent.

### Data Analysis

We analyzed survey responses using descriptive statistics to characterize clinicians’ perceptions of the AI-powered CDSS prototype. We summarized responses using means, SDs, and range values to assess overall trends and variability across items. Given the exploratory nature of the study and modest sample size, statistical analyses focused on identifying patterns in clinicians’ perceptions rather than inferential statistics.

We transcribed all think-alouds and interviews verbatim and imported them into NVivo (version 15; Lumivero) [45] for analysis. We used etic and emic qualitative coding to refine the provisional codes, with etic codes reflecting general domains and emic codes informed by the transcripts [46]. This coding approach allowed us to balance theory-driven and participant-driven perspectives. The etic codes provided a structured way to connect our analysis to the broader conceptual framework [35], ensuring that the codes aligned with established domains. At the same time, the emic codes captured participants’ own language, categories, and meanings, which were essential for representing their experiences interacting with the prototype.

Two members of our research team met to compare and discuss emergent codes and refined the codes and definitions to create a preliminary codebook. They then independently coded 20% of the transcripts to test for intercoder reliability (k=0.74) [47] before discussing and refining the codebook (Multimedia Appendix 4) further based on criteria such as data classification, coding category saturation, and coding regularities [48]. The final codebook resulted in 6 high-level categories: cognitive workload, expectancy, perception of AI, safety, situation awareness, and trust in AI. We organized 15 subcodes under these categories: 5 for cognitive workload, 2 for expectancy, 3 for situation awareness, and 5 for trust in AI. Two high-level categories (ie, perception of AI and safety) did not include any subcodes.

## Results

### Survey Results

Participants predominantly self-identified as women (13/16, 81.25%), with most participants (n=8, 50%) aged 35 to 44 years and identifying as either Black or African American (n=8, 50%) or White (n=7, 43.75%). Participants’ roles skewed toward prescribing clinicians, and most participants (n=10, 62.5%) had more than 5 years of experience in their role. Epic familiarity was high among participants. Nearly all participants (n=15, 93.75%) reported use of Epic, with 13 (81.25%) reporting daily use.

The postsurvey questions consisted of 6 constructs, which we directly adopted from previous literature [49-59] and customized for our study context (ie, 6 themes, detailed in Table S2 in Multimedia Appendix 2). Participants responded most positively to the usability and learnability items, with a theme mean (TM) 4.33 out of 5 (SD 0.66). Top-rated statements included, “It is easy to learn to use it” (TM 4.69, SD 0.48), “I can easily remember how to use it” (TM 4.56, SD 0.51), and “It is easy to use” (TM 4.50, SD 0.52), all with zero scores below 4. These responses indicate that participants read the HIV CDSS UIs as intuitive, even to nonearly adopters.

While participant responses to perceived effectiveness and satisfaction items were positive, there was a greater spread among them (TM 3.96, SD 0.84). Items related to satisfaction (“I am satisfied with it”) and willingness to reuse (“I will use it again”) scored 4.0 (SD 0.73) and 4.06 (SD 0.93), respectively. However, responses to items such as “It meets my needs” (TM 3.69, SD 0.79) and “It works the way I want it to work” (TM 3.63, SD 0.81) hint that workflows still need fine-tuning.

Responses to decision support quality and explainability and transparency hovered just under 4.0 (TM 3.83, SD 1.04) and showed the highest variance among the 6 themes. For instance, the item “I find the HIV CDSS UIs provide useful medical recommendations” had a TM of 3.75 with an SD of 1.18. At least one clinician strongly disagreed with the medical rationale. Thus, explanations likely need more depth and clarity. Trust signals were also found to be decent (overall TM 3.96, SD 0.77) but not unequivocal. For instance, both items “This system is trustworthy” and “The proposed HIV CDSS can be trusted” scored 3.88 (SD 0.72) and 3.81 (SD 0.83), respectively, underscoring the importance of reinforcing safety and validation.

Innovativeness was found to be the weakest area (TM 3.09, SD 0.80), indicating that participants, on average, did not view themselves as early adopters. However, responses within this construct showed internal variability, with some participants expressing strong confidence in and enthusiasm toward AI, while others positioned themselves as more cautious or reluctant adopters. This variation appeared to align with role and experience, as prescribing clinicians more frequently articulated skepticism, whereas participants in clinical support and care coordination roles expressed higher baseline trust in AI. In several instances, high trust ratings were not accompanied by detailed justification, suggesting that enthusiasm toward AI did not always reflect articulated technical understanding. Nonetheless, the positive usability scores suggest that late adopters still experienced smooth onboarding with the prototype.

Overall, clinicians reported the highest Likert scores for CDSS UI’s usability and learnability, followed by perceived effectiveness and satisfaction. This shows that the clinicians felt the UIs were intuitive and will help them effectively offer care to patients. Innovativeness had the lowest reported scores (Figure 2).

Survey responses suggest that while participants perceived the AI-powered HIV CDSS prototype as usable, learnable, and generally acceptable, clinicians’ confidence in its decision support quality, explainability, and trustworthiness was more conditional. To further examine how these perceptions emerge in practice, the next section describes findings from the think-aloud sessions and interviews, which provide deeper insight into how clinicians interpret AI predictions, negotiate trust, and articulate design expectations within the socioecological context of HIV care.

### Qualitative Data Results

We organized our qualitative data analysis results based on think-alouds and interviews in this subsection. The overall findings below span across multiple socioecological levels that shape clinician-AI interaction in HIV care. *At the individual level*, clinicians rely on familiar cognitive anchors, particularly viral load and CD4 lymphocyte count, to interpret AI-generated risk predictions. *At the interpersonal and organizational levels*, team-based workflows, role differentiation, and institutional constraints shape how predictions are evaluated, discussed, and acted upon. *At the societal level*, social determinants of health (ie, environmental conditions that affect a wide range of health, functioning, and quality-of-life outcomes and risks) emerge as critical contextual factors that clinicians routinely incorporate into their own predictive reasoning and expect AI systems to reflect. These levels illustrate how clinician-AI interaction is embedded within a broader socioecological system rather than occurring in isolation, helping to explain the conditional trust and variability observed in survey responses.

### Key Variables in Designing an AI-Powered HIV CDSS (RQ1)

Consistent with survey responses indicating moderate confidence in decision support quality, clinicians’ qualitative accounts revealed that their trust in AI predictions depended on whether the CDSS surfaced the same clinical and social variables they already use to assess patient risk. Prescribing clinicians (eg, physicians and nurse practitioners) and clinical support providers (eg, registered nurses and pharmacists), in particular, gravitated toward the same variables they routinely use to assess patient stability and risk, particularly viral load and CD4 lymphocyte count. These variables anchored their clinical reasoning and shaped how they interpreted the system’s predictions. During the think-aloud sessions, participants instinctively searched for these variables first, with P16 expressing, “I’m looking at CD4 lymphocyte count history for, okay, are we dipping back into risk,” and P12 explaining that she “went straight to the numbers, as far as the viral [load] and CD4 because that’s what I’m used to.”

For these participants, variables of viral load and CD4 lymphocyte count are not simply data points but cognitive cues that signal whether treatment is effective. As one participant explained:

*The viral load, that’s what we actually go into our labs [to see]. Those are the values that we look at when we are trying to decide if something is not going correctly with the treatment and the patient...because if the viral load increases above an undetectable level, then that can indicate that there either needs to be a treatment change or that there is a problem with the current treatment*.

[P10]

This example illustrates that specific variables are directly tied to risk detection and clinical action among clinicians. Thus, if a model omits or deemphasizes these variables, clinicians are less likely to trust or adopt its recommendations. Notably, while participants acknowledged that predicting viral failure is inherently complex and varies by patient, their decision-making consistently returned to these variables:

*I mean, [viral failure] is person dependent and things like that. And that’s the funny thing about prediction models. I mean, we always talk about my patient population as special and everybody’s a little bit different. But I mean, when I’m trying to snapshot my patient, I’d be thinking, recent CD4, recent viral load*.

[P15]

Thus, even when participants recognized patient-specific nuances, standardized clinical variables remained as their baseline orientation and the starting point for interpreting predictive outputs. As such, models should center these familiar variables while allowing flexibility for clinicians to incorporate additional contextual factors that further shape patient outcomes, particularly as prescribing clinicians, clinical support providers, and care coordination staff all cited social determinants of health as critical variables in their own predictive reasoning: “That’s how my non-AI model works. So, for [this model] to be better, it’s probably not enough to have just lab data in there” (P9). Participants such as P9 emphasized the importance of considering these social factors when assessing risk, describing how critical it is to “look at the whole picture, versus somebody just coming in and thinking they’re not just taking their meds.”

Among social determinants of health, participants commonly cited factors such as homelessness, socioeconomic status, and family support. Participants noted that housing instability is one of the most significant social factors predictive of viral failure among patients. P12 explained:

*As far as social aspects, one of the biggest factors is homelessness, because if they’re homeless, they’re not going to have a place to receive their meds. And so, that’s going to be an issue of sending their medications to the office, or where do you want us to send it? To the shelter? That’s one of the main factors that will a lot of times determine whether or not they’re going to be successful with taking their meds, especially with a new diagnosis.*

P15 further elaborated:

*You can’t make somebody take their HIV medicine if they’re worried about whether they have a roof over their head. It’s hard to prioritize. There are unhoused [patients] who are great at taking their medicine, and I don’t 100% understand how that dynamic works, but these are the factors that may play into potential for virologic failure or even connection to care*.

P12 and P15 demonstrate how clinicians view housing instability as a predictor of treatment interruption and subsequent viral failure. Clinicians framed homelessness not only as disrupting medication deliveries and refills but also as a competing priority that shapes a patient’s cognitive and emotional capacity to adhere to treatment.

Additionally, participants highlighted socioeconomic factors, particularly financial insecurity and insurance coverage, as central to predicting treatment continuity and viral suppression. As P7 explained:

*I think financial insecurity in some way could be a predictor of failure. You don’t come to your appointment because you’re afraid you can’t pay for it, so therefore you’re out of care.*

Similarly, P6 questioned:

*if insurance status could be something that’s built into the model...but the biggest thing for us is if patients can’t get their meds, they’re not going to be on their meds*.

These reflections demonstrate how clinicians view financial barriers not as peripheral context but as direct predictors of viral failure. In this sense, participants framed socioeconomic factors as necessary variables for an AI model to accurately reflect real-world patient trajectories.

Participants cited family support as another meaningful predictor. P1 described how support systems create divergent treatment pathways:

*Some people, when they get diagnosed, sometimes their family might disown them and that’ll kind of lead to other mental health, substance use type things. And then you have some who their families are great, and they actually help them and motivate them to take their medication.*

Here, family support functions as both a protective factor and a risk factor, with a supportive network having the potential to reinforce medication adherence and a lack of support potentially drawing patients off course.

These framings demonstrate that clinicians do not rely solely on clinical markers such as viral load and CD4 lymphocyte count when anticipating treatment outcomes. Instead, clinicians integrate social determinants of health into their own mental models of prediction, using these factors to anticipate viral failure. Thus, to be clinically meaningful, models must mirror this reasoning by weighing social determinants of health variables alongside clinical markers such as viral load and CD4 lymphocyte count.

### Barriers and Challenges to Designing CDSS (RQ2)

Survey findings showing mixed trust and explainability scores were reflected in clinicians’ qualitative accounts, which emphasized that hesitancy toward AI stemmed less from interface usability and more from concerns about clinical responsibility, workflow integration, and the pace of technological change. While these concerns were shared across roles, prescribing clinicians more often framed hesitancy in relation to decision-making authority and liability, whereas participants with clinical support and care coordination roles emphasized workflow integration and patient coordination.

Expanding on their survey responses, in which participants indicated that they do not view themselves as early adopters of AI technologies, participants revealed their hesitancy around AI in clinical contexts. Participants described CDSS as a critical part of patient care, and introducing emerging technology in this domain made them feel risky. As one participant noted, “These decision support tools are the most important thing that we can contribute to medical care now” (P6). In particular, participants’ concerns centered not on the idea of AI itself but on the pace of deployment and degree of trust participants felt they could place in automated predictions. P13 explained, “I’m inherently distrustful of AI. I think it’s being rushed out too fast, and I think it’s being shoved down our throats.” Here, hesitancy reflects a perceived misalignment between innovation and clinical reality. Participants wanted sufficient time to build confidence that AI can support their existing clinical judgment rather than override it completely.

Yet, this hesitancy was not an outright rejection of AI technologies. Instead, participants described conditional openness, grounded in the ability to evaluate AI against their own expertise, with participants such as P13 further explaining that they would be open to adopting AI over time if:

*I have the chance to use it in parallel with what I normally do, and you give me enough of a view of how it works, and I see that it helps me. I’d like to think that I’m willing to learn and better myself if it proves to be a useful tool, but I will be a little resistant.*

P1 expressed similar sentiments, saying:

*I’m still familiarizing myself with AI, and I know it gathers a lot of information that helps, is very accessible, but I think it’s definitely going to be helpful and guide helping the clinician to care for the patient*.

These responses demonstrate that trust must be earned over time. Adoption is dependent upon clinicians’ ability to verify usefulness and maintain agency in decision-making as they transition toward AI-supported workflows.

Explainability emerged as a foundation for trust during this transition. Participants consistently emphasized that explainability is essential for determining whether, and when, to rely on CDSS predictions. Without insight into how a system calculated its outputs, participants felt unable to justify clinical actions informed by AI. For instance, P16 expressed a need for “more clarity on how viral failure risk is calculated, how the definition is set,” noting that if she had a clearer “explanation for how the number was calculated, if I understood that better, I’d probably trust it more”.

In addition to concerns about model logic, participants also pointed to challenges in interpreting how data were visually presented, particularly along the visit timeline. The x-axis on the timeline used numbered indices rather than month-year time stamps, which made it difficult for some participants to contextualize when data points were recorded. As P16 noted:

*This visit date over here as I move to different time points...I’m not sure how to interpret the numbers for these dates of visits. It would probably feel more useful to me and make more sense if that were a month-year layout*.

Similarly, P15 questioned the temporal clarity of social determinants data, explaining:

*If it says injection drug use 2022, when was this data point pulled from? With the more social determinants of health, the stuff that people may not be getting every single visit, it’s worth potentially noting that that’s actually from 2020, etcetera.*

These comments suggest that explainability extends beyond algorithmic transparency to include clear temporal framing and data provenance within the interface itself.

This emphasis on transparency was particularly pronounced among prescribing clinicians responsible for therapeutic decision-making. Particularly, these participants cited that understanding the exact variables contributing to the system’s predictions would enable them to take more targeted actions, especially around social factors, allowing them to “work on some of these things that predict the failure. That’s why the explanation is so important. If I see half of [a patient’s] risk is because he can’t get anywhere, then I’ll fix that more than the other [variables]” (P9). For some participants, breakdowns of variable weighting would serve as a bridge between AI reasoning and clinicians’ existing mental models of risk, enabling them to engage in the most appropriate interventions. P13 explained:

*If you say patient has a 67% chance of failure, and you say 20% of that is drug abuse and 15% of that is history of incomplete adherence and 15% of that is homelessness, right then, okay, maybe I can have social work see if we can get them housing*.

Other participants, however, preferred less granular explanations, emphasizing efficiency rather than detail, citing, “I’m not so interested in the nitty gritty of this got higher weight and this got lower weight” (P15).

Notably, explainability was situational, with participants wanting greater transparency when the system’s outputs differed from their own predictions or clinical instincts:

*If my feeling does not agree with the AI generator, I might click on it and go to say, okay, why is the AI predictor saying this is high risk and I would say they’re more moderate risk or low risk? But as long as my feeling looking at that individual patient matched, I don’t know that I would click to have a better explanation.*

[P15]

Here, P15 highlights a key dynamic of incorporating AI in clinical decision-making, in that trust in AI is conditional and comparative. Clinicians do not interpret AI predictions in isolation but rather evaluate them against their own assessments. Explainability, therefore, becomes a mechanism not only for understanding model behavior but for negotiating authority between human and algorithmic judgment. When AI predictions align with clinical reasoning, explanations feel unnecessary to clinicians. When these predictions diverge, interpretability becomes essential for reconciling differences. In this sense, explainability acts as a safeguard that preserves clinicians’ decision-making agency during their gradual adoption of AI in clinical decision-making.

### AI Design Features (RQ3)

To meaningfully contribute to clinical decision-making, participants emphasized that an AI-driven CDSS must provide not only a risk assessment but clinically actionable next steps. Risk prediction alone does not reduce cognitive burden or enhance their judgment, particularly given their confidence in identifying risk from familiar indicators. As P13 explained:

*At the end of the day, I need something to intervene on. I don’t need a risk assessment, because of my risk assessment that I can just look at his viral load.*

Here, P13 illustrates that the value of AI lies not in duplicating clinicians’ existing expertise but in advancing care beyond risk identification. Participants expressed that they often already have “a general sense of when [failure] is going to happen,” leading them to want support for “the next step. It’s all well and good to have a risk, but a risk needs an intervention. You need to be able to react to the data” (P13). Thus, actionable guidance is a foundational requirement in AI-powered CDSS. Participants envisioned recommendations that would directly address variables contributing to failure, allowing clinicians to “address some of those situations” (P1). While prescribing clinicians emphasized therapeutic intervention, clinical support roles framed actionability in terms of coordinating and operationalizing care. These participants highlighted a core expectation of an AI-powered CDSS, where predictions imply a responsibility to intervene. In this sense, clinicians wanted the system to surface not only what is going wrong, but where targeted intervention could prevent failure:

*If we can identify, we know there are risks, so we can identify the percent risk, what do you do? How do you fix it? If you could really figure it out, then you could probably help [patients] better.*

[P7]

Participants also stressed that interventions must extend beyond clinician support to patient education, reinforcing adherence and shared decision-making. Participants framed visual, explanatory features as necessary for improving outcomes. As P9 stated:

*If the ultimate goal of the system is to make us have less virological failure, [you need to] get it to somebody who has the most interest in this, which is the patient. It should be the patient.*

Here, patient-facing tools shift the CDSS from a purely clinical tool to a collaborative one that helps build patient agency. This emphasis on patient education was particularly pronounced among participants in care coordination roles. For instance, P5 highlighted how the visualizations are:

*Something that we can show patients as well, because sometimes talking to them, you kind of have to meet people where they are. Everybody is different. So, just talking to them like this, we can show them, hey, this is your viral load. This is what’s going on when you don’t take your medicine. So, visuals are better for a good bit of our patients.*

These interactions reveal that clinicians already rely on explanations and visuals to build understanding and adherence. Incorporating similar educational elements could strengthen the system’s usefulness by operating at both the clinical and behavioral layers of care.

## Discussion

### Principal Findings

This study makes a theoretical contribution by significantly extending Choudhury’s [35] conceptual sociocognitive framework for clinician-AI interaction through a socioecological and systems-oriented lens grounded in an empirical HIV care field study. While prior work emphasizes individual cognitive factors such as trust, expectancy, and cognitive workload, our findings demonstrate that clinician-AI interaction in HIV care is shaped by layered social, organizational, and structural contexts that dynamically influence interpretation, reliance, and action. By empirically grounding these dynamics, we advance a prescriptive framework that clarifies how AI-powered CDSS should be designed to support clinician reasoning, preserve professional agency, and account for the team-based and stigmatized nature of HIV care. In doing so, this work moves beyond merely identifying factors that influence AI acceptance to articulating how these factors can be operationalized through design and lived clinical practices.

Our findings indicate that extending sociocognitive frameworks for clinician-AI interaction requires moving beyond a focus on individual cognition to account for the socioecological systems in which clinical decision-making is embedded. In HIV care, clinician reasoning unfolds across interconnected levels, including individual cognitive processes, team-based workflows, organizational constraints, and broader social and structural conditions that shape patient adherence and access to care. These levels are not independent; rather, they dynamically interact to influence how AI outputs are interpreted, trusted, and translated into action over time.

**Figure 4.**
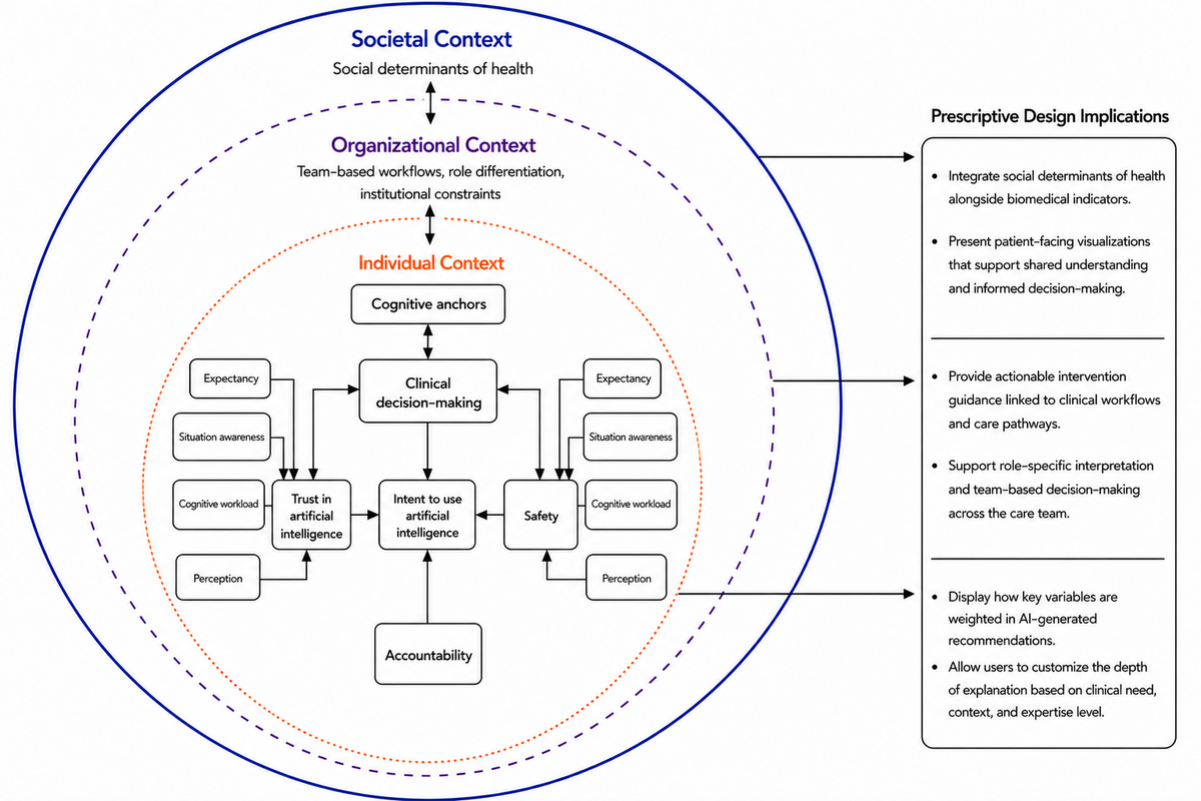
Extended socioecological sociocognitive framework for clinician-AI interaction in HIV care. Choudhury’s [35] original conceptual framework (center) is embedded within individual, organizational, and societal layers identified through empirical findings. The framework highlights prescriptive design implications for AI-powered CDSS implementation in HIV care environments. Adapted from Choudhury [35], which is published under Creative Commons Attribution 4.0 International License [60].

This study examined how HIV care clinicians assess the usefulness, trustworthiness, and design of an AI-powered CDSS for predicting virologic failure. Our findings, derived from think-aloud interactions with the CDSS and follow-up interviews, demonstrated that clinicians’ existing cognitive models of patient risk remain anchored in familiar biomedical indicators, particularly viral load and CD4 lymphocyte count, while also incorporating social determinants of health as critical predictors of treatment continuity. For AI-powered CDSS to be meaningfully integrated into their clinical reasoning process, participants emphasized that such systems must complement rather than replicate their expertise, providing targeted insights and actionable intervention strategies that extend beyond what clinicians already recognize. Additionally, findings revealed that trust in AI is conditional and earned over time among clinicians, particularly through explainability, clinical alignment, and demonstrable improvements in patient care. In doing so, our findings extend Choudhury’s [35] descriptive framework to offer a prescriptive, design-oriented framework (Figure 4), illuminating how AI systems should be built to operationalize trust and enhance, not disrupt, the cognitive and clinical practices that underpin HIV care. In this sense, our findings move from which variables shape trust and intent to use AI in clinical settings to how AI systems should be designed to operationalize that trust and intent in practice.

### Centering Clinician Cognitive Anchors in AI-Powered CDSS Design

Findings demonstrate that clinicians’ interpretations of AI-powered risk predictions remain grounded in the same cognitive anchors they routinely use to assess treatment stability in practice, namely viral load and CD4 lymphocyte count. Participants did not approach these variables as isolated data points; rather, they used them as familiar “red flags” that indicate whether current treatment is effective and whether intervention is needed. These findings align with longstanding cognitive research showing that clinicians rely on internalized cues (ie, informational signals that direct attention and activate abstract knowledge structures during clinical reasoning) [61]. Through years of clinical experience, these cues become expert heuristics that enable decision-making under conditions of bounded rationality. Thus, when AI systems center these trusted indicators, the system can support rather than disrupt this cue-based reasoning.

Notably, clinicians also described social determinants of health, such as housing instability, financial insecurity, and family support, as key predictors of medication adherence and, ultimately, viral suppression. Participants incorporated these contextual factors into their mental models of prediction, demonstrating that cognitive cues in HIV care extend beyond biomedical markers to encompass the lived realities of patients. For AI systems to meaningfully support clinical judgment, they must integrate both biomedical and social factors that clinicians view as critical drivers of treatment outcomes. In practice, social determinants must be systematically captured, whether through structured EHR fields or screening instruments, and incorporated as weighted predictors alongside biomedical indicators. Clinicians alluded to the lack of information on critical social determinants of health, such as income, housing, living conditions, and access to care that were not captured in model training or displayed in the prototype. Such information is only available to clinicians and case managers that interact with patients regularly and remain unaccounted for in many medical databases. Study participants felt that these data points must be inputted into the model in some capacity to improve risk prediction. Within an AI-powered CDSS interface, these factors should be surfaced transparently, allowing clinicians to see how biomedical and social contributors interact to shape predictions. For example, risk estimates could visually distinguish between laboratory-driven risk (eg, viral blips) and structurally driven risk (eg, housing instability), enabling clinicians to align interventions accordingly. In this way, social determinants move from peripheral context to operationalized variables that directly inform both risk stratification and recommended care pathways. Failing to reflect this complexity risks producing predictions that feel incomplete or clinically irrelevant, thus threatening acceptance and trust in the AI system.

This connection between cognitive cues and model interpretability is crucial for ensuring situational awareness, which refers to clinicians’ understanding of the current and evolving status of a patient in a dynamic environment [62]. If AI systems elevate unfamiliar variables or obscure familiar ones within complex algorithmic outputs, they risk creating information misalignment. In this sense, when familiar clinical cues are buried or deemphasized, an AI-powered CDSS may inadvertently demand more cognitive work instead of alleviating it.

Participants were most receptive to AI insights when the model foregrounded the cues they already use to monitor treatment progress. In contrast, they were skeptical when recommendations were grounded in variables they could not easily place within their established mental model of HIV care. These dynamics highlight the interplay between situational awareness and trust. Participants trusted outputs that aligned with their cognitive anchors and viewed misaligned outputs as needing greater justification.

These findings add nuance to mixed prior evidence regarding how trust and situational awareness relate. Some research suggests that situational awareness shapes clinicians’ willingness to use AI [63], while other work proposes that trust is a precursor to situational awareness [64]. Here, our findings underscore how models must be designed to surface the specific information that clinicians deem most clinically diagnostic. These findings also speak to fundamental differences in how clinicians and AI systems reason. Clinicians are boundedly rational, relying on cue-based heuristics that simplify complex situations [65]. AI systems, in contrast, aggregate a far broader array of statistics without the ability to selectively filter irrelevant cues [66]. Thus, as AI systems become more complex and “accurate,” they risk growing less aligned with human thought processes. An AI-powered CDSS that ignores clinicians’ cognitive anchors, therefore, threatens situational awareness and ultimately trust.

### Trust as Conditional and Comparative

Findings revealed that clinicians’ trust in the AI-powered CDSS was conditional and determined through ongoing comparison with their own clinical judgment. Participants expressed openness to using AI but emphasized that trust in the system would be earned through demonstrated alignment with clinical reasoning and through gradual, parallel use, rather than an abrupt replacement of their expertise and established workflows. These findings align with expectancy theory [37], which suggests that clinicians will engage with AI systems when they believe the system will improve performance without incurring additional effort or risk.

Explainability emerged as foundational for building clinician trust. Participants described needing insight into why patients were flagged as high risk to determine whether and how to act on the AI prediction. This finding echoes prior work establishing explainability as a primary mechanism for supporting trust and promoting reliance in clinical settings [4,53,67], particularly as trust is central to safe and effective human-AI collaboration [68]. In high-stakes domains, such as HIV care, clinicians must be able to justify AI decisions to themselves and to patients, making lack of transparency a significant barrier to adoption [55].

At the same time, our findings illustrate that explainability is not universal across clinicians. Participants most often sought explanations only when AI predictions diverged from their own clinical knowledge. In these instances, explainability served as a tool for preserving professional authority, which is consistent with previous work demonstrating that trust emerges over time [68] through calibrated collaboration between humans and AI rather than through blind confidence in AI systems [53]. Explanations, thus, become a form of authority negotiation, where explainability supports clinician agency when AI challenges their existing mental models.

These findings add nuance to often simplified assumptions that explainability universally improves trust. Previous research has argued that explainability alone cannot resolve misalignments in goals and logic between AI and clinicians [61]. Here, participants framed explainability as useful only when it clarified model-identified risks connected to variables that meaningfully map onto real-world clinical action, especially social determinants of health. These findings suggest that trust requires more than just transparency. Rather, trust depends on contextual relevance and interpretive alignment. As such, trust is not simply confidence in algorithmic accuracy, particularly as even highly accurate AI systems may not earn clinicians’ full trust if they perceive it as disconnected from the human-centered values of health care [69]. Rather, trust is determined by how closely AI systems align and strengthen clinicians’ own judgment, particularly in situations of uncertainty or disagreement. In this sense, explainability functions as a key mediator, enabling clinicians to assess when to rely on AI predictions and when to lean more heavily on their existing expertise.

Additionally, as suggested by previous work [70], explanations must be customizable to clinicians’ goals, roles, and workflows, given that information needs vary. Thus, AI systems cannot use a single explanatory approach. Our participants demonstrated this variability with some participants expressing a need for more granular breakdowns of contributing risk factors, while others prioritized efficiency and surface-level explanations. These findings indicate that explainability must be designed as a flexible, sociocognitive process that supports different modes of engagement [55].

### Shifting From Risk Identification to Actionable Support

Findings demonstrate that risk prediction alone is insufficient for supporting clinicians’ decision-making. The prototype CDSS surfaced risk scores and contributing variables, leaving the translation from prediction to intervention largely to clinician interpretation. As clinicians already rely on familiar indicators and internalized heuristics to identify patients at elevated risk [65], findings indicate that an AI-powered CDSS that simply flags high-risk patients without further guidance introduces an additional layer of interpretation, thus increasing, rather than alleviating, cognitive effort. These findings align with bounded rationality principles [71] in clinical reasoning, wherein clinicians rely on fast, intuitive reasoning and selectively attend to a limited set of cues to reach sufficiently effective decisions [65].

Thus, actionability must be a central component of an AI-powered CDSS. As illustrated by participants, predictions implicitly convey a responsibility to intervene, meaning that AI systems must connect identified risks to meaningful and context-sensitive next steps for clinicians to act upon. Recent work underscores the importance of transparency for intervention development [72], suggesting that revealing how an AI system generates its predictions can help guide clinicians toward appropriate interventions. Such transparency shifts AI systems as merely predictive systems into collaborators that aid clinicians in targeting the drivers of risk.

To be effective, however, intervention guidance must be tailored to patient context and delivered in ways that support communication and team-based decision-making. Prior research has shown that generic or poorly contextualized recommendations underline perceived usefulness of AI systems [73]. In this sense, generic recommendations risk missing essential variables, such as family support or housing instability, that clinicians deeply understand but may not be reflected in structured clinical data, like underlying EHRs. As such, AI systems lacking this contextual personalization will fail to address the “whole person” [73], making them poorly suited to replace the nuanced conversations clinicians rely on to build care plans. Designing actionable AI-powered CDSS features that integrate both clinical and social variables is therefore critical for creating ecologically valid HIV decision support that translates into meaningful patient outcomes.

### Study Strengths and Limitations

This study had several limitations. First, we recruited participants from a single HIV clinic within one health care system in South Carolina, which may reflect local workflows, norms, and patient populations that differ in other settings. As we recruited participants within a specific geographic context, regional factors, such as resource availability, health care infrastructure, and population-specific social determinants of health, may shape clinicians’ experiences and expectations of AI-powered CDSS in ways that are not fully transferable to other settings. Second, the risk-level prediction shown in our prototype did not include a timeline. The absence of such a horizon affects the actionability of the provided insight, especially in longitudinal settings such as HIV care where distinction between short-term and long-term impact is critical.

Furthermore, some limitations arose from the methodological design choices of this study. While think-aloud data captured participants’ immediate engagement with the CDSS, anticipatory reasoning during the interviews may have shaped how some participants described the system and its predictions. As such, some reflections may represent clinicians’ broader mental models of AI rather than interpretations derived exclusively from direct interaction with the system’s explainability features.

Finally, the Explanation view shown in the prototype (Figure 3) contained raw, unformatted outputs from the LIME model. While this provided participants with an under-the-hood peek into the risk level assigned for the specific patient, this could have negatively impacted participants’ understanding of the prototype, particularly those participants who are not familiarized with medical databases and variable naming conventions, such as novice clinicians.

### Conclusions

These findings collectively demonstrate that clinician-AI interaction in HIV care is best understood as a socioecological and dynamic process rather than a series of isolated cognitive judgments. Clinicians interpret AI outputs through established cognitive anchors, calibrate trust through ongoing comparison and explanation, and expect AI systems to support concrete actions that account for social and structural realities of patient care. By empirically grounding these processes across individual, organizational, and societal levels, this work significantly extends Choudhury’s [35] sociocognitive framework from a descriptive account of clinician-AI interaction to a prescriptive, systems-oriented framework for AI-powered CDSS design. Such an extension is particularly critical in chronic and stigmatized care contexts such as HIV, where effective decision support must align not only with clinician cognition but also with the socioecological conditions that shape patient outcomes.

## Supplementary material

10.2196/91620 Multimedia Appendix 1 .Example interfaces of patient use cases in the AI-powered CDSS prototype.

10.2196/91620 Multimedia Appendix 2 Study participants’ demographics and professional characteristics and post-survey constructs. .

10.2196/91620 Multimedia Appendix 3 Interview guide.

10.2196/91620 Multimedia Appendix 4 Coding scheme and definitions for themes identified in clinician feedback on the AI-powered HIV CDSS prototype.

## Abbreviations

CDSS: Clinical Decision Support System
EHR: electronic health record
LIME: Local Interpretable Model-Agnostic Explanations
ML: machine learning
RF: Random Forest
RQ: research question
TM: theme mean
UI: user interface
USC: University of South Carolina
