# Supplementary material for: Advancing Human-Centered AI in Clinical Decision Support: Sociocognitive Human-in-the-Loop Study in HIV Care
Source: J Med Internet Res. 2026 Jul 31;28:e91620. doi: 10.2196/91620 (PMC13427062; doi:10.2196/91620)
Supplement: Multimedia Appendix 1 [file jmir-v28-e91620-s001.docx]

### **Multimedia Appendix 1: Supplemental Figures**


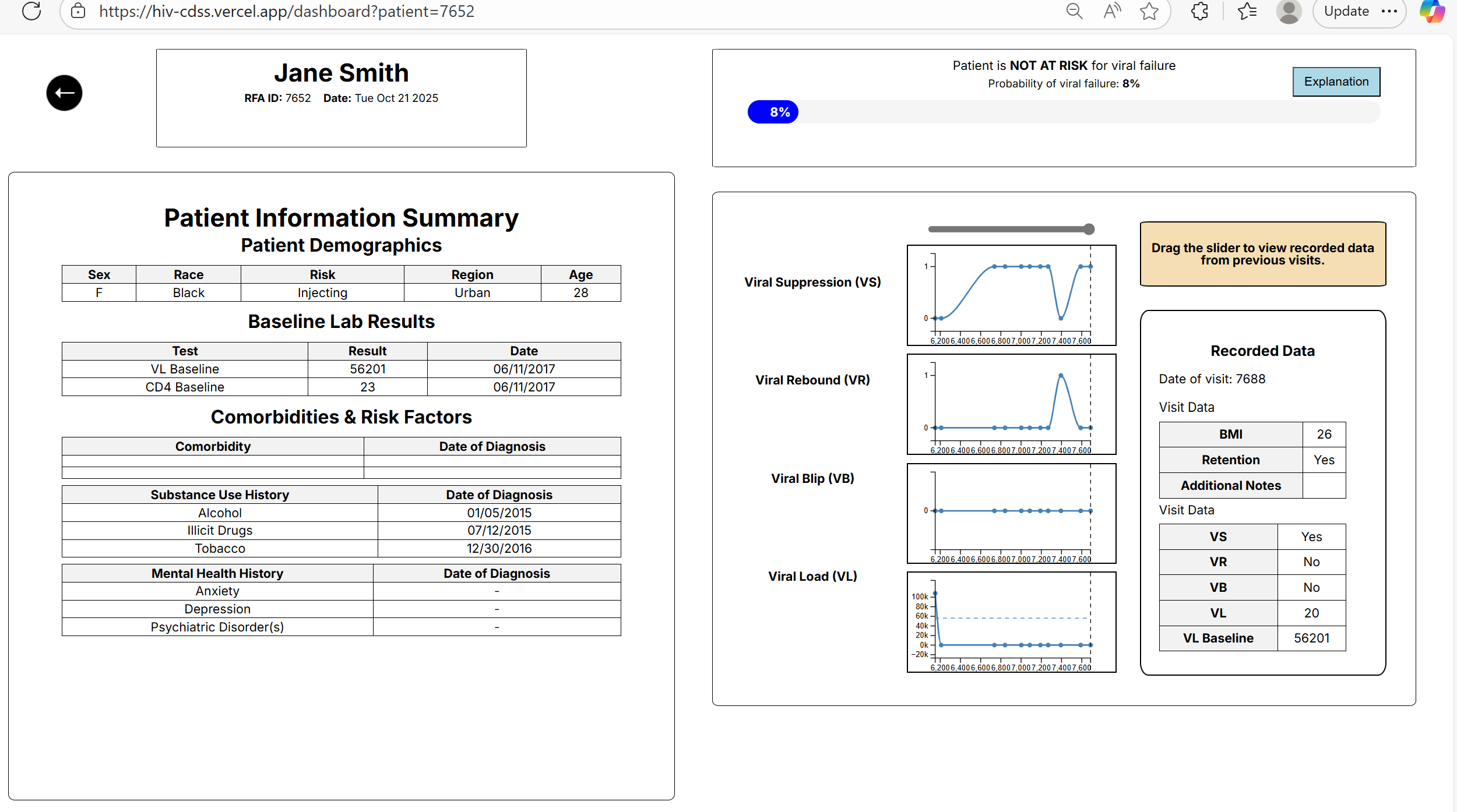


Figure S1. Example interface of a low-risk HIV patient use case in the AI-powered CDSS prototype, showing patient demographics, baseline lab results, comorbidities and risk factors, longitudinal trends in key viral indicators, and a low predicted risk of viral failure displayed with supporting recorded clinical data


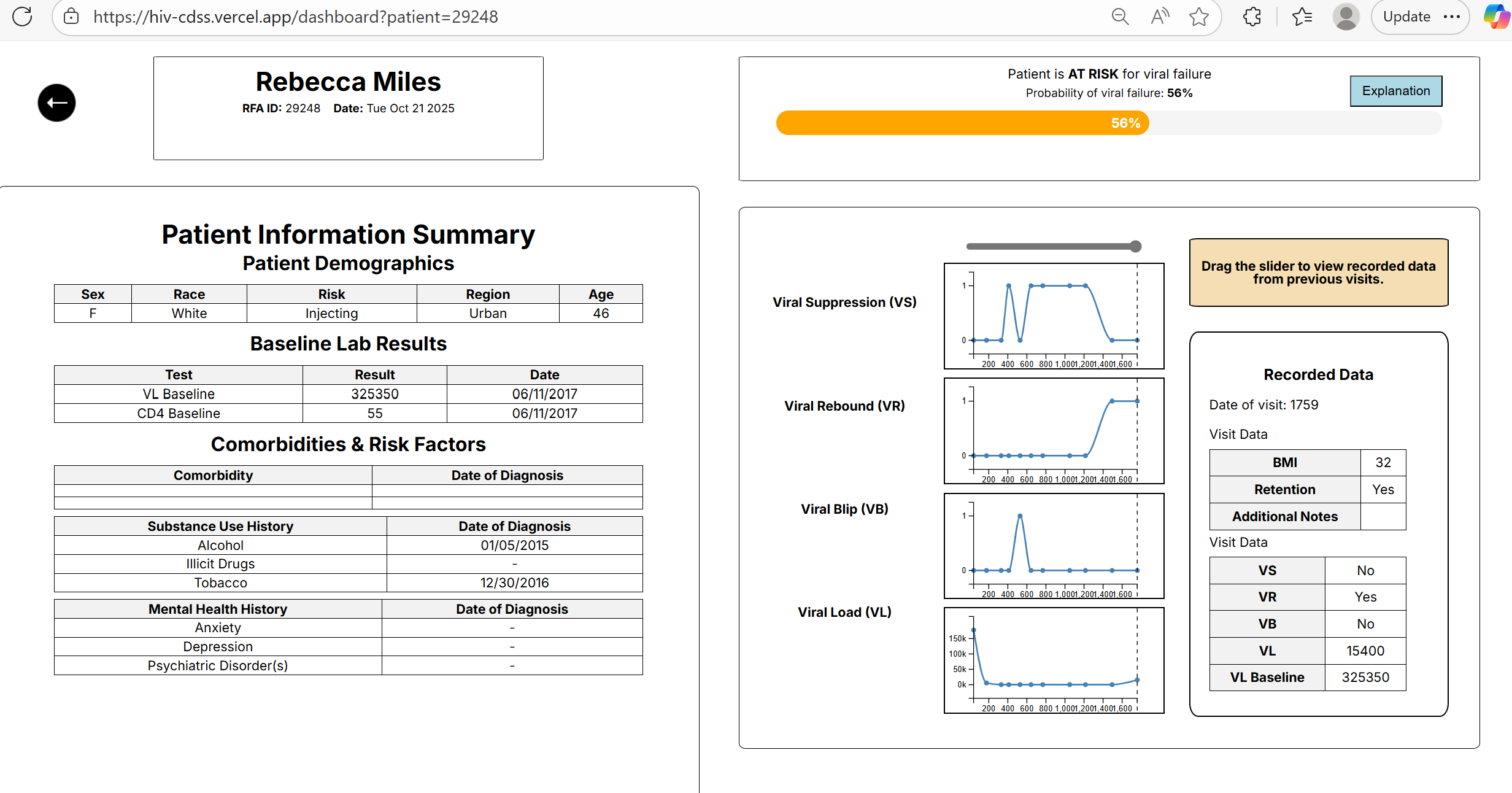


Figure S2. Example interface of a medium-risk HIV patient use case in the AI-powered CDSS prototype, showing patient demographics, baseline lab results, comorbidities and risk factors, longitudinal trends in key viral indicators, and a moderate predicted risk of viral failure displayed with supporting recorded clinical data


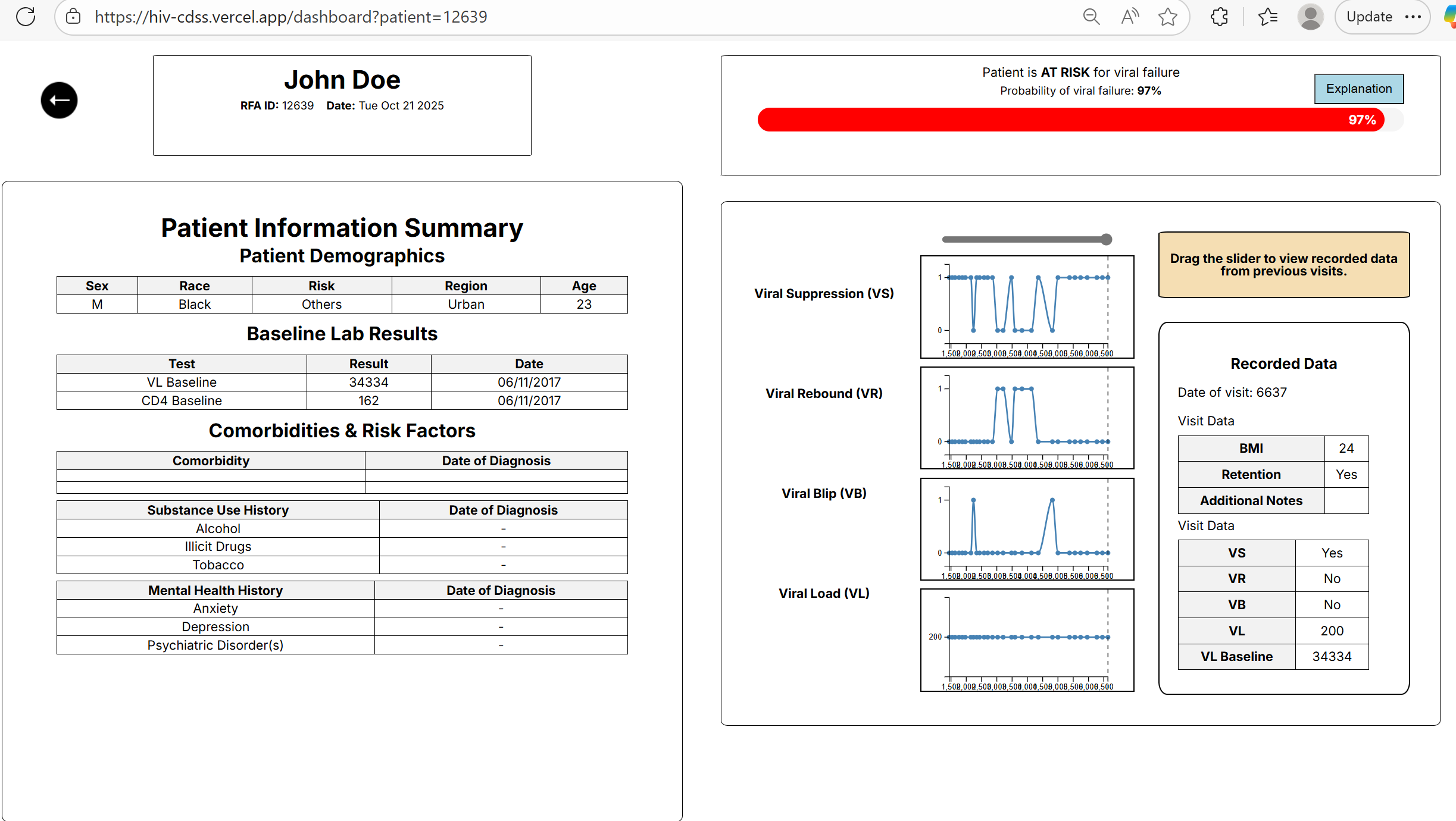


Figure S3. Example interface of a high-risk HIV patient use case in the AI-powered CDSS prototype, showing patient demographics, baseline lab results, comorbidities and risk factors, longitudinal trends in key viral indicators, and a high predicted risk of viral failure displayed with supporting recorded clinical data
