# Supplementary material for: Advancing Human-Centered AI in Clinical Decision Support: Sociocognitive Human-in-the-Loop Study in HIV Care
Source: J Med Internet Res. 2026 Jul 31;28:e91620. doi: 10.2196/91620 (PMC13427062; doi:10.2196/91620)
Supplement: Multimedia Appendix 2 [file jmir-v28-e91620-s002.docx]

### **Multimedia Appendix 2: Supplemental Tables**

| **Participants** | **Job Title** | **Age** | **Race/Ethnicity** | **Gender** | **Roles and Responsibilities** | **Years of Experience** | **AI Trust Level** | **AI-CDSS UI Suggestions** |
| --- | --- | --- | --- | --- | --- | --- | --- | --- |
| **P1** | Medical Assistant | 35-44 | Black or African American | Female | Handles the administrative side of work, checks for viral load and CD4, verifies medications, and interviews and schedules patients | Longer than 10 years | Not much idea about AI, trusts 75% | Best if everything is on a single-screen interface |
| **P2** | Infectious Disease Pharmacist (PharmD) | 25-34 | White | Female | Carries out reviewing tasks such as medication access (e.g., prior authorizations), following up on appeals, medication management for providers, improving patient suppression rates, and filling prescriptions for patients | 1-3 years | Would trust the system based on the clarity | Create a separate tab beside the patient's name |
| **P3** | Social Worker | 25-34 | Black or African American | Female | Provides social support, addresses HIV patients’ basic needs, educates patients, and helps with medication tracking | 1-3 years | Fully trusts AI | Needs flexibility to move the risk bar and its visual display |
| **P4** | Clinical Counselor | 45-54 | Black or African American | Female | Helps HIV patients with behavioral health screening, counseling, and scheduling appointments | 1-3 years | Mostly trusts AI | Prefers resource-based interface features |
| **P5** | Outreach Supervisor | 35-44 | Black or African American | Female | Locates people, such as those who are unhoused, and spends time with them, helping them schedule appointments. Checks with their insurance, assists patients with referrals, helps people who are out of care, and especially helps prison- and jail-released patients | 5-10 years | Strong belief, 100% trusts AI | Prefers to have ways to print files, like a download button of patient information with different colors on the explanation bar |
| **P6** | Infectious Disease Pharmacist (PharmD) | 25-34 | White | Male | Works more in the decision-making process by advising patients to clinicians, checking chart reviews, helping patients with adherence challenges, and connecting them with social workers and medication help | 1-3 years | Trusts based on validation of its use | Add patients’ full history and more explanations on the parameters of risk bar graph |
| **P7** | Infectious Disease Pharmacist (PharmD) | 35-44 | White | Female | Checks HIV patients for adherence, medication tracking, transitions of care, like checking for outpatient IV antibiotics for patients, second-line overseer, makes sure things are flowing appropriately. Conducts meetings that involve reviewing the costs, funds, and dollars required to run audits and associated implementations | Longer than 10 years | Trust depends on time | Further break down the time to a month, a day, or a year due to current confusing positions that are difficult to understand based on the inputs.  Add out of care, which would be a good predictor, like financial insecurity, as well as risk acquisition instead of risk injection |
| **P8** | Jail Linkage & Prison Case Manager | 55-64 | Black or African American | Female | Brings patients from jail and provides case management, including medication and enrollment in applicable benefit programs, such as the South Carolina app program. Also provides medication to the jail and, in the case of prison, takes care of patients with post-release follow-up and links them to care | Longer than 10 years | Trusts AI | Add lab appointments tab |
| **P9** | Physician (MD) | 55-64 | White | Male | Prescribes medication for HIV patients. Works collaboratively with nurses and case managers. Responsible for diagnosis, treatment planning, and comprehensive patient management. Mainly focuses on clinical decision-making | Longer than 10 years | Trust is based on model validation | Make anxiety and depression more granular within the model and add a  second button on the date of diagnosis |
| **P10** | Registered Nurse (RN) | 45-54 | Black or African American | Female | Assists HIV doctors in prescribing medications and checks with virals. Supports physician | 1-3 years | Trusts AI | Add patient compliance, such as medication as prescribed, and missing appointments |
| **P11** | Social Worker | 45-54 | Black or African American | Female | Gets patient demographic information and history, as well as assists with referrals and compliance checks | Longer than 10 years | Believes that trust increases once you start working with AI | Add appointments |
| **P12** | Nurse Practitioner (NP) | 35-44 | Black or African American | Female | Checks patients' compliance, attending to physicians ' new and returning patients | 1-3 years | Trusts AI but would like to go back and check if the information is accurate | Add a clinical notes section as a template and current antiretroviral regimen |
| **P13** | Physician (MD) | 35-44 | White | Male | Takes responsibility to make clinical decisions, coordinates with medical staff, and works in a team | 5-10 years | Distrusts AI, which demands experimentally validated models, and prefers an actionable intervention | Prefers plain English explanations with a dictionary, simple visuals, and remove redundant binary graphs |
| **P14** | Physician (MD) | 35-44 | Asian | Female | Delivers comprehensive clinical care to patients with HIV, including evaluation, diagnosis, and prescription of antiretroviral and supportive medications. Leads treatment planning and medical oversight, ensuring continuity of care across the care team | Longer than 10 years | Generally trusts AI | Add patient education materials |
| **P15** | Physician (MD) | 35-44 | White | Female | Provides medical management for individuals living with HIV, including prescribing antiretroviral therapy and other necessary medications. Works closely with nursing staff and case managers to coordinate care and monitor treatment adherence. Responsible for diagnosis, ongoing assessment, and individualized treatment planning to support optimal health outcomes | 5-10 years | Believes that trust is dependent on quality of training data: “garbage in, garbage out” | Include data on medication and appointment history |
| **P16** | Nurse Practitioner (NP) | 35-44 | White | Female | Conducts independent practice and prescribes, assesses, and educates patients. Cares for HIV patients who are underserved, uninsured, and insured | Longer than 10 years | Thinks that trust would increase over time and with reliable outputs | Contextualize substance use data |

P: Participant

Table S1. Demographic and professional characteristics of participants (n = 16), including job title, gender, role and responsibilities, years of experience, level of trust in AI, and suggested improvements to the AI-powered CDSS prototype

| **Constructs/Themes** | **Questions** |
| --- | --- |
| Usability & Learnability (e.g., [58]) | 'It is easy to use'  'Using it is effortless'  'I can use it without written instructions'  'I can easily remember how to use it'  'It is easy to learn to use it'  'I quickly became skillful with it'  'I became familiar with the HIV CDSS UIs very quickly' |
| Perceived Effectiveness & Satisfaction (e.g., [55, 57]) | 'It is useful'  'It makes the things I want to accomplish easier to get done'  'It meets my needs'  'It works the way I want it to work'  'I am satisfied with it'  'I will use it again'  'The HIV CDSS UIs will add value to HIV treatment'  'The HIV CDSS UIs can help me make medical decisions faster'  'The HIV CDSS UIs can help me to make better treatment choices regarding viral failure' |
| Decision Support Quality (e.g., [56, 58]) | 'I like the viral failure alerts provided by the HIV CDSS UIs'  'The alerts make clinical sense as provided'  'I find that the HIV CDSS UIs provides useful medical recommendations'  'I am convinced by the medical suggestions that the HIV CDSS UIs recommended to me'  'The proposed HIV CDSS through its UI demo provides sufficient medical recommendations for me to make a good medical decision' |
| Explainability & Transparency (e.g., [50, 53-54]) | 'The HIV CDSS UIs explain why medical recommendations were recommended to me'  'I understand why the medical recommendations were recommended to me'  'The information provided by the HIV CDSS UIs helped me understand the decision-making process of the system'  'The visualization provided by the HIV CDSS UIs helped me understand the decision-making process of the system'  'I understand how the system will assist me with the decisions I have to make'  'It is easy to follow what the system does'  'I recognize what I should do to get the information I need from the system the next time I use it' |
| Trust (e.g., [49, 51-52]) | 'I believe the information that the HIV CDSS UIs provide me'  'This system is trustworthy'  'The proposed HIV CDSS through its UI demo can be trusted' |
| Innovativeness (e.g., [48]) | 'I usually keep an eye on emerging technology products'  'I always try out new technology products earlier compared to others' |

Table S2. Post-survey constructs and corresponding items used to evaluate participants’ perceptions of the AI-powered HIV CDSS prototype
