# Supplementary material for: Advancing Human-Centered AI in Clinical Decision Support: Sociocognitive Human-in-the-Loop Study in HIV Care
Source: J Med Internet Res. 2026 Jul 31;28:e91620. doi: 10.2196/91620 (PMC13427062; doi:10.2196/91620)
Supplement: Multimedia Appendix 3 [file jmir-v28-e91620-s003.docx]

### **Multimedia Appendix 3: Interview Guide**

1. Do the overall current AI-powered HIV CDSS prototype user interfaces (UIs) make sense to your practice?
   1. How, if at all, do they represent your current routine at your practice?
   2. Are there any challenges or barriers in your clinical practice that these UIs do not address or represent clearly?
2. What potential benefits or improvements to your current routine do you see these HIV CDSS UIs offering in the future?
   1. How could these UIs evolve to better support your work?
3. Now, I’m interested in hearing your thoughts on how the AI in the CDSS system generated the results:
   1. For instance, what information do you think the algorithm considered in its analysis to generate that specific result?
   2. What information do you think an algorithm should prioritize or consider in its analysis?
   3. Now, the inverse of that, what information do you think an algorithm shouldn’t prioritize or consider in its analysis?
4. How useful did you find the explanation in the HIV CDSS UIs?
   1. What stood out to you the most about the explanation?
5. Which UI design was the most confusing to you?
   1. What made it confusing?
6. How did the current UI designs meet your expectations in providing medical recommendations?
   1. What aspects of the HIV CDSS worked well for you when receiving medical recommendations?
   2. Were there any areas where the medical recommendations fell short of what you expected? If so, can you explain?
   3. How could the design be improved to make the medical recommendations more helpful for your practice?
7. Now, I want to talk more about your interaction with the UIs.
   1. What aspects of the interaction felt easy or intuitive to you?
   2. Were there any parts where you felt unsure or struggled?
   3. How could the design be improved to make interacting with the UIs more seamless for you?
8. We plan to implement this set of UIs in a real AI-powered HIV clinical decision support system as the next phase of development. If we were to implement these UIs in Epic tomorrow, how much would you trust them?
   1. What specific factors or features would make you trust the system? Can you give examples of what would increase your trust in using them?
9. Where in your current Epic system would you suggest that we implement the HIV CDSS to best match your routine workflow?
   1. In what ways would this integration be used and adopted by your clinical practice?
   2. What concerns or barriers do you see with integrating this system?
10. Our last question for today: What is something you think is important for researchers like ourselves working on AI-powered clinical decision support systems to know?
    1. Finally, was there anything I didn’t ask you today that I should have or something we didn’t cover that you think we should have?
