# Supplementary material for: Advancing Human-Centered AI in Clinical Decision Support: Sociocognitive Human-in-the-Loop Study in HIV Care
Source: J Med Internet Res. 2026 Jul 31;28:e91620. doi: 10.2196/91620 (PMC13427062; doi:10.2196/91620)
Supplement: Multimedia Appendix 4 [file jmir-v28-e91620-s004.docx]

### **Multimedia Appendix 4: Coding scheme and definitions for themes identified in clinician feedback on the AI-powered HIV CDSS prototype**

| **Name** | **Description** |
| --- | --- |
| **Cognitive Workload** | The mental burden or strain imposed on the clinician by integrating the AI system into their work environment. |
| Alignment with Existing Workflow | Integration must align with existing workflows. Features outside of existing workflows are less likely to be used. |
| Integrated Information | The consolidation of key variables, such as demographics, comorbidities, lab results, risk factors, and risk probabilities, on one screen reduces the need to navigate through multiple screens or systems. |
| Just Enough Information | Keep information minimal so to include only key variables and time trends as the focus (e.g., six-month trends for viral loads). |
| Redundant Information | Redundancy among graphs and variables should be reduced to minimize cognitive burden. |
| Visualizations | Risk indicators and trend graphs provide a more intuitive experience compared to scrolling through raw data. |
| **Expectancy** | As an operationalization of motivation, expectancy suggests that the probability of a clinician deciding to use an AI-derived decision is dependent on the degree to which the clinician believes that this specific behavior will lead to an intended positive outcome (e.g., patient safety). |
| Action-Oriented | The clinician needs a clear “do next,” such as specific recommendations, not only long, technical explanations. |
| Patient Education | The clinician emphasizes the importance of educating patients to improve adherence, such as having patient-friendly sub-tabs of the information that can be shared with patients (e.g., “what-if” simulations that show how behavior changes, such as appointment adherence, would lower risk). |
| **Perception of AI** | The clinician's overall views regarding AI, specifically their perception of patient safety (risk) and their perception of AI accountability (who is responsible for a flawed AI system or incorrect output). |
| **Safety** | If the clinician misinterprets the AI output, they may be misled into making wrong clinical decisions, which puts patient safety at risk. Safety, generally, involves minimization of both risk and epistemic uncertainty. |
| **Situation Awareness** | The clinician's fundamental understanding of the current clinical situation, which is affected by how relevant information is presented. |
| Clinical Data | The clinician notes that they trust variables they already use (e.g., viral load) and will adopt a system that uses clinical data consistent with their existing practices. |
| Contextualized Data Points | The clinician expresses a need to move beyond a binary classification (i.e., yes/no) of a specific data point to a more open-ended response (e.g., frequency and type of illicit drug use, visit patterns such as missed appointments) or to provide background on the data sources (e.g., when the data point was entered). |
| Social Determinants of Health | The clinician notes the importance of considering social determinants of health into predictions of risk (i.e., non-medical factors that influence health outcomes, such as education or access to transportation). |
| **Trust in AI** | Trust in technology is generally associated with reliability and performance. For AI systems, trust comes from confidence that the system will err extremely rarely, achieved through system training, exhaustive testing, safety measures, and standards. |
| Explainability | The clinician expresses their willingness to trust in the system when the reasoning and other factors are clear with explanations. |
| Mistrust in Prediction | The clinician states that the risk prediction goes against their clinical expertise or “gut feeling.” |
| Model Validation | The clinician requires model validation metrics, such as the percent of consistency between prediction and actual outcome or requires confirmation that the prediction correlates with existing lab outcomes (e.g., the clinician verifies the results). |
| Recognition of Complex Variables | The clinician recognizes that some variables are highly dynamic, interact with each other, and are difficult to access, track, and evaluate (e.g., social determinants of health) and thus cannot be factored into an algorithm, which may influence the accuracy of predictions. |
| Capturing Complex Variables | The clinician offers suggestions to add brief questionnaires for complex variables (e.g., health literacy, social support) or to store these variables as structured fields. |
